# Effector T cell chemokine IP-10 predicts cardiac recovery and clinical outcomes post-myocardial infarction

**DOI:** 10.3389/fimmu.2023.1177467

**Published:** 2023-06-22

**Authors:** Kateryna Sopova, Simon Tual-Chalot, Matthias Mueller-Hennessen, Nikolaos I. Vlachogiannis, Georgios Georgiopoulos, Moritz Biener, Marco Sachse, Andrey Turchinovich, Maria Polycarpou-Schwarz, Luke Spray, Eleni Maneta, Karim Bennaceur, Ashfaq Mohammad, Gavin David Richardson, Aikaterini Gatsiou, Harald F. Langer, Norbert Frey, Kimon Stamatelopoulos, Joerg Heineke, Daniel Duerschmied, Evangelos Giannitsis, Ioakim Spyridopoulos, Konstantinos Stellos

**Affiliations:** ^1^ Translational and Clinical Research Institute, Vascular Biology and Medicine Theme, Faculty of Medical Sciences, Newcastle University, Newcastle Upon Tyne, United Kingdom; ^2^ Department of Cardiology, Angiology, Haemostaseology and Medical Intensive Care, University Medical Centre Mannheim, Medical Faculty Mannheim, Heidelberg University, Mannheim, Germany; ^3^ Department of Cardiology, Royal Victoria Infirmary (RVI) and Freeman Hospitals, Newcastle Upon Tyne Hospitals National Health Service (NHS) Foundation Trust, Newcastle Upon Tyne, United Kingdom; ^4^ German Centre for Cardiovascular Research (DZHK), partner site Heidelberg/Mannheim, Mannheim, Germany; ^5^ Department of Cardiovascular Research, European Center for Angioscience (ECAS), Heidelberg University, Heidelberg/Mannheim, Germany; ^6^ Biosciences Institute, Vascular Biology and Medicine Theme, Faculty of Medical Sciences, Newcastle University, Newcastle Upon Tyne, United Kingdom; ^7^ Department of Internal Medicine III, Cardiology, University Hospital Heidelberg, Heidelberg, Germany; ^8^ Department of Clinical Therapeutics, Alexandra Hospital, National and Kapodistrian University of Athens School of Medicine, Athens, Greece; ^9^ Department of Cardiovascular Physiology, European Center for Angioscience (ECAS), Medical Faculty Mannheim, Heidelberg University, Mannheim, Germany

**Keywords:** IP-10, myocardial infarction, T cells, prognostic value, lymphocytes, heart failure, remodeling, cardiac MRI

## Abstract

**Background and aims:**

Preclinical data suggest that activation of the adaptive immune system is critical for myocardial repair processes in acute myocardial infarction. The aim of the present study was to determine the clinical value of baseline effector T cell chemokine IP-10 blood levels in the acute phase of ST-segment elevation myocardial infarction (STEMI) for the prediction of the left ventricular function changes and cardiovascular outcomes after STEMI.

**Methods:**

Serum IP-10 levels were retrospectively quantified in two independent cohorts of STEMI patients undergoing primary percutaneous coronary intervention.

**Results:**

We report a biphasic response of the effector T cell trafficking chemokine IP-10 characterized by an initial increase of its serum levels in the acute phase of STEMI followed by a rapid reduction at 90min post reperfusion. Patients at the highest IP-10 tertile presented also with more CD4 effector memory T cells (CD4 T_EM_ cells), but not other T cell subtypes, in blood. In the Newcastle cohort (n=47), patients in the highest IP-10 tertile or CD4 T_EM_ cells at admission exhibited an improved cardiac systolic function 12 weeks after STEMI compared to patients in the lowest IP-10 tertile. In the Heidelberg cohort (n=331), STEMI patients were followed for a median of 540 days for major adverse cardiovascular events (MACE). Patients presenting with higher serum IP-10 levels at admission had a lower risk for MACE after adjustment for traditional risk factors, CRP and high-sensitivity troponin-T levels (highest vs. rest quarters: HR [95% CI]=0.420 [0.218-0.808]).

**Conclusion:**

Increased serum levels of IP-10 in the acute phase of STEMI predict a better recovery in cardiac systolic function and less adverse events in patients after STEMI.

## 1 Introduction

Coronary artery disease (CAD) is the single largest cause of mortality worldwide, directly accountable for 9.4 million of deaths every year (1). Acute ST-segment elevation myocardial infarction (STEMI) is one of the most severe manifestations of CAD which may lead to left ventricular systolic dysfunction and subsequent heart failure. Although the management of STEMI has significantly advanced in recent years, 6-month mortality remains elevated at 13%, while a third of surviving patients develop heart failure over the following 5-8 years after MI (2–4). The persistence of left ventricular (LV) systolic dysfunction a few months after STEMI is a strong predictor of morbidity and mortality among STEMI patients (5, 6). Therefore, there is an urgent need to identify early predictors of LV function recovery and of cardiovascular outcomes to identify the residual risk in patients with STEMI and to correctly risk stratify these patients in order to offer a tailored individualised approach of patient management and disease treatment.

While early primary percutaneous intervention can limit myocardial infarct size in patients with STEMI, reperfusion triggers an intense inflammatory response, characterised by mobilisation of leukocytes in the blood followed by an abundant leukocyte infiltration in the infarcted region (7). Although inflammation is a determinant of heart function after STEMI in experimental acute myocardial infarction (AMI) models, the contribution of adaptive immunity in human cardiac repair remains poorly understood. We have previously reported that circulating levels of total T lymphocytes in the acute phase of STEMI predict long-term survival in these patients (8). However, the underlying mechanisms remain unknown.

Preclinical studies have reported distinct roles of the two main subsets of T lymphocytes, CD4^+^ and CD8^+^, in acute ischemia. CD4^+^ T cells have been shown to exert cardioprotective effects in a preclinical model of AMI by acquiring a T-regulatory (Treg) phenotype and suppressing the local inflammatory response (9, 10). On the other hand, the role of CD8^+^ T cells post-myocardial infarction remains unclear. From one side, several studies suggested that the presence of CD8^+^ T-cells in the heart may lead to myocardial necrosis, cardiac dysfunction and poor survival post-MI (11–13). However, another study reported that CD8^+^ T cells may play both a beneficial and a detrimental role in cardiac remodeling, depending on the stage of left ventricular remodeling. In specific, the latter study reported that CD8^+^ T cells may initiate the removal of necrotic tissue and decrease the incidence of cardiac rupture in a murine model of MI after permanent ligation of the left anterior descending coronary artery (14). At a later stage, CD8^+^ T cells induce cytotoxic effects on healthy cardiomyocytes and enhance neutrophil and macrophage-mediated inflammation leading to left ventricle dilation and reduced cardiac function (14). The CD8^+^ T cell responses may be beneficial in the acute phase of AMI in the regulation of scar formation but may be maladaptive in the long term. Understanding the temporal and spatial regulation of these immune cells is of utmost importance for the identification of residual risk and risk stratification of patients with STEMI and the development of novel immunomodulatory therapeutic strategies against LV remodelling post-MI.

Interferon-γ-inducible protein-10 (IP-10, also known as CXCL10) is a chemokine secreted by innate immune cells (monocytes, neutrophils, natural killer (NK), and NKT-like cells) and sentinel cells of the innate immune system, such as the endothelial cells and fibroblasts, which are dedicated to respond to cytokines and damage-associated molecular pattern molecules emitted upon tissue injury (15, 16). IP-10 elicits its biological effects by binding to its receptor CXCR3 and is integral for recruiting activated (effector) T cells to the sites of tissue inflammation (15). IP-10 levels are known to be elevated in animal models of experimental myocardial infarction and in patients with coronary heart disease (17–19). Preclinical studies suggest that IP-10 is integral for CXCR3-positive immune cell recruitment to the ischemic myocardial tissue and regulation of reparative response following experimental AMI in mice (19, 20). However, its translational value in myocardial repair processes in patients with STEMI remains unknown. Herein we tested the hypothesis that serum IP-10 levels on admission may predict recovery of left ventricular systolic function and future cardiovascular outcomes in patients after STEMI.

## 2 Methods

**Figure 1:**
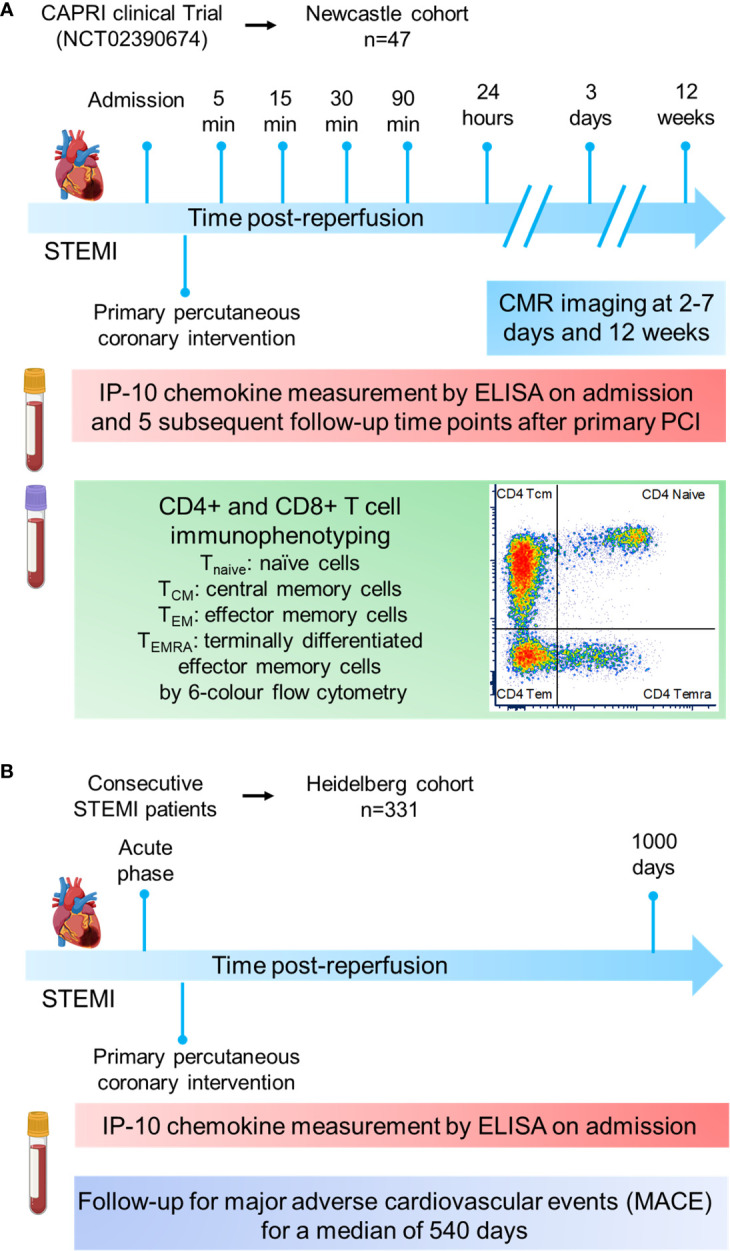
Cohort study design. **(A)** The Newcastle cohort is derived from the CAPRI clinical trial (NCT02390674). Blood from 47 STEMI patients was collected at admission and at sequential time-points post-reperfusion (5, 15, 30, 90min and 24h) to measure IP-10 by ELISA and proceed to CD4^+^ or CD8^+^ immunophenotyping by flow cytometry. Cardiac MRI scans were obtained 3 days as well as 12 weeks post‐myocardial infarction. **(B)** The Heidelberg cohort constited of 331 STEMI patients. Blood was collected at admission to measure IP-10 by ELISA. Patients were followed up for major adverse cardiovascular events for a median of 540 days.

The **Figure 1** introduces in a flow chart the two patient cohorts and the methodology applied in this study.

### 2.1 Population and design

#### 2.1.1 Cardiac recovery post-STEMI (Newcastle) cohort

The first cohort consisted of 47 out of 52 patients with available serum probes from the double-blind, randomised controlled trial (Cyclosporin to Reduce Reperfusion Injury in Primary PCI Clinical Trial; “CAPRI”, NCT02390674) (demographics in **Table 1**, named as Newcastle cohort) (21, 22). All participants had to present with STEMI and undergo primary percutaneous coronary intervention (primary PCI), be at least 18 years of age, and present within 6 hours of the chest pain onset. The culprit coronary artery had to be a major coronary artery with a diameter of at least 3 mm and had to be proximally occluded at the time of admission identified by coronary angiography. Exclusion criteria for this trial were patients presenting with immunological disorders, cardiogenic shock, unconscious patients, evidence of coronary collaterals to the infarct area, open culprit coronary artery at the time of angiography, previous myocardial infarction or thrombolytic therapy, sensitivity to cyclosporin or egg, peanut or soya-bean proteins, treatment with any compound which may modify blood concentration of cyclosporin, known renal or liver insufficiency, uncontrolled hypertension (>180/110 mmHg), female patients either currently pregnant or of child-bearing age with no current contraceptive use (verbal diagnosis), use of investigational study drug within the previous 30 days, life expectancy of less than one year due to non-cardiac illness, or patients with contraindication to cardiac MRI. Following angiography, participants were randomised in a 1:1 ratio to either cyclosporin (n=26) or control (saline, n=26) using a blocked allocation (permuted random blocks of variable length) system immediately before reperfusion implemented using Newcastle Clinical Trials Unit’s online randomisation service. Cyclosporin (Sandimmun^®^, Novartis) was given as an intravenous infusion dissolved in saline (maximum concentration 2.5mg per millilitre) of 2.5mg per kilogram of body weight (maximum total amount 250 mg). After successful infusion, the coronary wire was advanced and the culprit lesion crossed, followed by revascularisation. Patients underwent primary PCI according to standard guidelines. All patients received a loading dose of two antiplatelet drugs and heparin before PCI. Use of thrombus aspiration and/or glycoprotein IIb/IIIa inhibition were used according to the operator’s discretion. Zotarolimus- or everolimus-eluting stents were implanted.

**Table 1:** Patient characteristics of the cardiac recovery post-STEMI (Newcastle) cohort according to baseline IP-10 tertiles.

IP10- tertiles (baseline)	Total cohort (n=47)	Lowest (n=16)	Middle (n=16)	Highest (n=15)	P-value*
Demographics					
Male sex	39 (83.0)	13 (81.3)	13 (81.3)	13 (86.7)	0.899
Age (years)	65.5 ± 10.8	61.0 ± 12.0	67.5 ± 10.7	68.1 ± 8.6	0.123
BMI, kg/m^2^	27.7 ± 6.1	27.0 ± 8.4	27.0 ± 4.6	29.2 ± 4.5	0.526
Comorbidities					
Diabetes mellitus	4 (8.5)	2 (12.5)	0 (0)	2 (13.3)	0.322
Hypertension	8 (17.0)	3 (18.8)	1 (6.3)	4 (26.7)	0.311
Hyperlipidemia	4 (8.5)	2 (12.5)	0(0)	2 (13.3)	0.322
eGFR (mL/min)	81 (23)	87 (32)	75 (22)	78 (23)	0.337
Myocardial infarction characteristics					
Onset-to-reperfusion (min.)	196 ± 91	212 ± 105	189 ± 98	185 ± 69	0.676
admission hs-cTnT (ng/L)	122 ± 208	200 ± 300	74 ± 155	88 ± 95	0.096

Continuous variables are presented as mean ± SD or median (IQR) and categorical variables as absolute count (valid percentage).

*P-value is derived from one-way ANOVA or the non-parametric Kruskal-Wallis test for continuous variables when non-normally distributed and from the chi-square test for categorical variables.

BMI, body mass index; eGFR, estimated glomerular filtration rate; hs-cTnT, high-sensitivity cardiac troponin T.

### 2.1.2 Prospective STEMI for MACE (Heidelberg) cohort

Patients presenting to the emergency department of Heidelberg University Hospital, Germany with a working diagnosis of acute coronary syndrome (ACS) were consecutively recruited from the 9^th^ June 2009 to the 10^th^ May 2014 (23). Patients were retrospectively adjudicated, and analysis was restricted to a final diagnosis of STEMI with available follow-up for major adverse cardiovascular events (MACE) and available blood samples at admission, leaving 331 for measurement of serum IP-10 (demographics in **Table 2**, named as Heidelberg cohort). In brief, MI was diagnosed using high-sensitivity (hs) cardiac troponin T (cTnT) in accordance with the universal MI definition (24). Only patients with complete follow-up information were screened for eligibility. Follow-up was performed by telephone contact and/or questionnaires sent by email or land mail. Information on mortality events was further obtained by the local residents’ registry. Follow-up was executed at different time points since this is a registry with repetitive adjudication of diagnoses and events. All main analyses were performed for a maximum follow-up time of 1000 days (median 540 days) and vital status was censored at this time point. This time point was selected because it provided the optimal balance between the longest possible follow-up and missing information. Specifically, medical records were reviewed and evaluated regarding the incidence of MACE, which were defined as CVD death, AMI and performance of coronary revascularisation procedure. CV death was defined as death resulting from MI, sudden cardiac death, death due to heart failure, stroke and other CVD causes (25). Coronary revascularisation procedures included percutaneous coronary intervention or coronary artery bypass grafting. The research protocol did not interfere with the management of the study patients. Treatment decisions were made by the attending cardiologists.

**Table 2:** Descriptive characteristics of the prospective STEMI cohort for MACE (Heidelberg cohort) by highest vs lower quarters of baseline IP-10 levels.

	Total cohort (n=331)	Lower quartiles (n=249)	Highest quartile (n=82)	P-value*
Demographics				
Male sex	243 (73.4)	192 (77.1)	51 (62.2)	0.010
Age (years)	64 (21)	62 (20)	67 (21)	0.009
Cardiovascular risk factor				
Diabetes mellitus	72 (22.6)	54 (22.4)	17 (21.8)	1.000
Arterial hypertension	227 (70.5)	172 (70.2)	55 (71.4)	0.887
Hyperlipidemia	162 (52.1)	119 (50.4)	43 (57.3)	0.353
History of MI	50 (15.8)	36 (15.1)	14 (17.9)	0.593
History of heart failure	49 (16.0)	30 (12.7)	19 (26.8)	0.009
Presenting features				
Heart rate (bpm)	78 (24)	78 (22)	80 (26)	0.127
SBP (mmHg)	146 (31)	145 (31)	147.5 (31)	0.902
Admission hs-cTnT (ng/L)	91 (610)	90 (631)	100.5 (506)	0.856
C-reactive protein (mg/dL)	4.8 (13.2)	4.2 (11)	6.85 (23)	0.035
eGFR (MDRD)	83.8 (36.3)	86.5 (35)	71.8 (31)	0.001
GRACE score	150 (46)	144 (48)	165 (47)	<0.001
Cardiovascular outcomes				
Combined endpoint **	105 (31.8)	88 (35.5)	17 (20.7)	0.014

Continuous variables are presented as median (IQR) and categorical variables as absolute count (valid percentage).

*Comparison between the highest vs lower IP-10 quartiles. P-value is derived from one-way t-test or the non-parametric Mann Whitney U test for continuous variables when non-normally distributed and from the Fisher’s exact test for categorical variables.

**Composite of all-cause death, myocardial infarction, stroke, resuscitation and revascularisation (percutaneous coronary angiography, coronary artery bypass graft).

CVRF, cardiovascular risk factor; SBP, systolic blood pressure; hs-cTnT, high-sensitivity cardiac troponin T; eGFR, estimated glomerular filtration rate; GRACE, Global Registry of Acute Coronary Events; MI, myocardial infarction.

### 2.2 Cardiac magnetic resonance imaging

In the Newcastle cohort, CMR imaging was obtained with a Siemens Avanto 1.5 Telsa MRI scanner, using a phased array body coil combined with a spine coil at 2-7 days post-reperfusion, and repeat CMR at 12 ± 2 weeks was obtained to assess myocardial function improvement by calculating change in LV ejection fraction (ΔLVEF) (21). All images were obtained during breath-holding. Localiser images were acquired as well as axial black blood HASTE images to define the anatomy. Cine images of the heart in 2, 3 and 4 chamber views were obtained using a steady-state free precession pulse (SSFP) sequence (repetition time [TR]: set according to heart rate, image matrix 144x192, echo time (TE): 1.19ms, flip angle: 80°). T2 weighted STIR (short inversion time [TI] inversion recovery) images were then obtained in the same projections, using a black-blood segmented turbo spin-echo technique (TR according to heart rate, TE 47ms, flip angle 180°, TI 140ms, image matrix 208x256). Further sequential end-diastolic STIR images were acquired along the short-axis of the heart, covering the full extent of the left ventricle in parallel slices (each 8mm with 0mm gap). Corresponding short-axis SSFP cine images were then obtained to allow quantification of chamber volumes and function. Intravenous Gadobutrol contrast (Gadovist, Bayer Schering Pharma AG, Berlin, Germany) was administered at a dose of 0.1mmol/kg. After 10 minutes short-axis end-diastolic late gadolinium enhancement images (in corresponding locations to cine and STIR images) were obtained using an inversion recovery (IR) segmented gradient echo sequence (TR: according to heart rate, TE: 3.41ms, flip angle: 25°, image matrix: 196x256). The inversion time (TI) for late gadolinium enhancement imaging was selected in order to null normal myocardium (giving it a dark appearance), and adjusted throughout acquisition (increased approximately every second slice) to maintain nulling. As previously described, all analyses were performed using validated cardiac MRI analysis software (cvi42, Circle Cardiovascular Imaging Inc., Calgary, Canada) (8). LV volumes and mass were determined using the short axis SSFP cine images, following the determination of the longitudinal extent of the chamber by cross-referencing with the 4 and 2 chamber images (8). Epicardial and endocardial borders were traced automatically on each end-systolic and end-diastolic short-axis cine frame with manual correction where necessary, allowing automated calculation of left ventricular mass, dimensions and ejection fraction (LVEF), as previously described and validated (21, 22).

### 2.3 Multicolour flow cytometry

A 6-colour assay was used to measure the expression of CD3, CD4, CD8, CD45RA, CCR7 and CX_3_CR1 in blood samples as previously described and validated (26). All flow cytometric measurements were carried out on fresh blood within hours of collection. 50μL fresh blood aliquots from every time point were added to their corresponding labelled 5mL Falcon tubes. A cocktail of antibodies (Mouse Anti-Human CD3, 555333, BD Biosciences; Mouse Anti-Human CD4, 560768, BD Biosciences; Mouse Anti-Human CD8, 555366, BD Biosciences; anti-human CD197 (CCR7), 353208, BioLegend; CD45RA (L48) PE- Cy7, 337186, BD Biosciences; anti-human CX_3_CR1, 341610, BioLegend) were added to the sample. After a 20-minute incubation, 1mL of fresh lysis buffer was added to each mixture. Following another 20-minute incubation, the sample was washed and run through a BD FACSCanto-II Flow Cytometer (339473, BD Biosciences) using the BD FACSDiva acquisition software. Cells were gated on CD3^+^ T-lymphocytes and 20,000 events were recorded allowing for representative data. We gated T-lymphocytes (CD3^+^) first into CD4^+^ and CD8^+^ T cells, then into four subsets of each by expression of CCR7, the chemokine receptor for CCL19/21, and CD45RA, as previously described (27). Four sub-populations of CD4^+^ and CD8^+^ T cells were investigated: naïve (T_Naive_), central memory (T_CM_), effector memory (T_EM_) and terminally differentiated effector memory cells (T_EMRA_). No significant amount of non-viable cells (<1%) was detected. Percentages and absolute count of T lymphocyte subtypes determined in peripheral blood on admission, at 5, 15, 30 and 90 minutes and 24 hours post-reperfusion are reported.

### 2.4 Blood measurement

Levels of IP-10 were measured in serum samples using a commercial enzyme-linked immunosorbent assay (ELISA) (R&D Systems, Minneapolis, MN, USA), which is commonly considered the gold-standard assay for analyte quantification in clinical research (28). In the Newcastle cohort, IP-10 concentration was measured retrospectively on admission and at various sequential time-points post-reperfusion (5, 15, 30, 90min and 24h). In the Heidelberg cohort, IP-10 levels were retrospectively measured at admission. The intra- and inter-assay coefficients of variance of the ELISA measurements were reported to be less than 3.6% and 6.7% respectively, and the minimum detectable concentration of human IP-10 was 4.46 pg/mL (R&D Systems). All measurements were performed by experienced personnel who were blinded to patients’ characteristics.

### 2.5 Ethics statement

Favourable ethical opinions were received for the CAPRI study (National Research Ethics Committee North-East – Newcastle and North Tyneside 2; REC reference: 14/NE/1070), and the Heidelberg cohort (21, 23). CAPRI received a clinical trial authorisation from the Medicines and Healthcare products Regulatory Agency (MHRA). All STEMI/PPCI patients analysed retrospectively had provided written informed consent to their clinical data included in a database of PPCI procedures for research. All studies used complied with the principles laid out in the declaration of Helsinki, and written informed consent was obtained from all participants.

### 2.6 Statistical methods

Data are presented as mean ± standard deviation or median (interquartile range) for continuous variables and count (percentage) for nominal variables. Normal distribution of all continuous variables was graphically assessed by distributional plots, including histograms, symmetry plots and normal-quantile plots, as well as by Kolmogorov-Smirnov and Shapiro-Wilk tests. Baseline characteristics were compared with analysis of variance or with the non-parametric Kruskal-Wallis test for continuous variables and with the Pearson’s chi-square test for nominal variables. Pair-wise comparisons (highest vs lower IP-10 quarters) of continuous variables were performed by Dunnett’s (parametric) or Dunn’s (non-parametric) multiple comparisons test, while chi square test was used to compare categorical variables between contrast groups. In the Newcastle cohort, the primary endpoint of our study was a change in LVEF at 12 weeks post-STEMI versus LVEF at 2-7 days (median: 3 days) post-STEMI (ΔLVEF). Linear regression was used to examine the association of IP-10 levels with ΔLVEF, as a cardiac repair marker, after adjustment for age, sex, body mass index (BMI), diabetes mellitus (DM), arterial hypertension, hyperlipidemia, estimated glomerular filtration rate (eGFR) and admission high sensitivity cardiac troponin T (hs-cTnT) as biologically plausible variables affecting cardiac repair.

In the Heidelberg cohort, the primary endpoint was a composite of all-cause death, AMI, stroke, resuscitation and revascularisation (percutaneous coronary angiography, coronary artery bypass graft). Nelson–Aalen curves were generated to depict the cumulative incidence of outcomes. Subjects were censored at the time of the first event. Log-rank test was used in the Nelson Aalen curves figure. Cox proportional-hazards models were used to examine the association between IP-10 levels and the primary outcome. Associations are presented as Hazard Ratio (HR) with 95% confidence intervals (CI). Statistical analysis was performed with SPSS v. 26 (IBM) and STATA v. 13 (StataCorp). All tests were two-tailed. We deemed statistical significance at P<0.05.

#### 2.6.1 Power calculations

*A priori* calculations showed that a sample size of 47 subjects would be adequately powered at 0.85 level to detect a minimum of 3% increase in ΔLVEF post STEMI between patients at highest and lower tertiles of IP-10 by the non-parametric Mann Whitney test. Dispersion measures for ΔLVEF were calculated based on previously published method (29). Type I error was set at 0.05. Power calculation was performed with G Power 3.1.

## 3 Results

### 3.1 Dynamic biphasic regulation of serum IP-10 levels in STEMI

**Figure 2:**
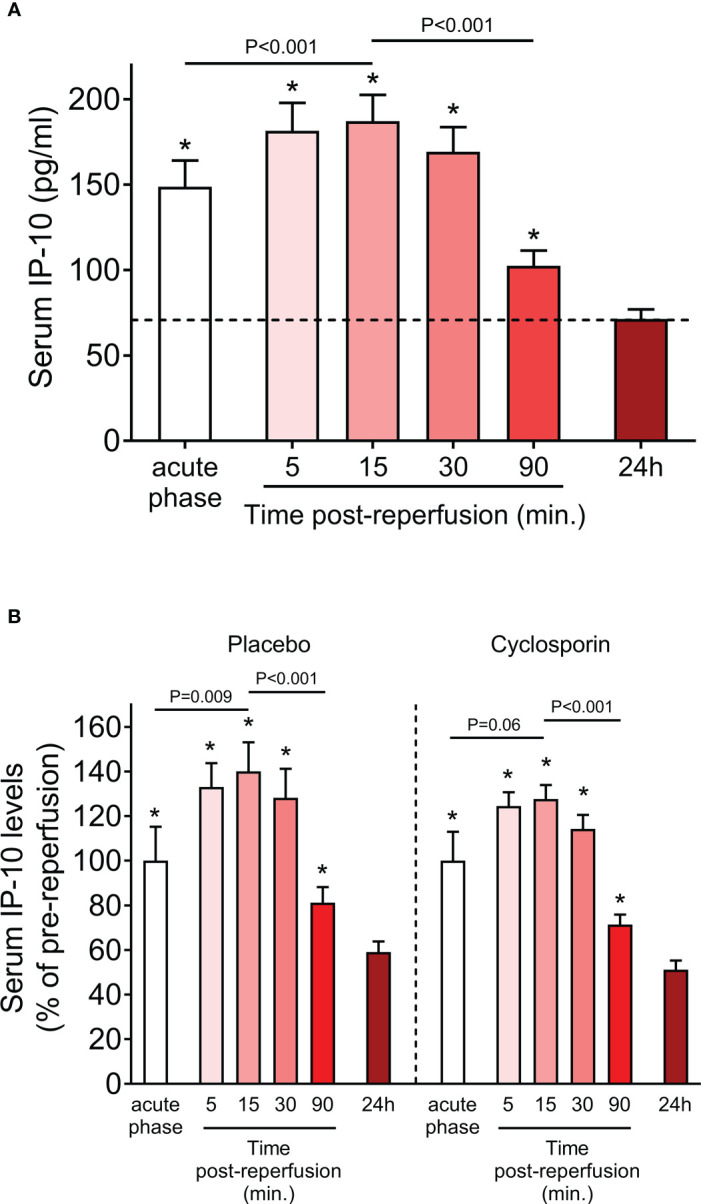
Serum IP-10 levels in STEMI patients. **(A)** Serum IP-10 kinetics at admission (acute phase) and at sequential time-points post-reperfusion (5, 15, 30, 90min and 24h) in 47 patients with ST-segment elevation myocardial infarction (STEMI). ^*^P<0.001 for each pair-wise analysis compared to the 24h time-point. (Wilcoxon signed-rank test). **(B)** Serum IP-10 kinetics at admission (acute phase) and at sequential time-points post-reperfusion (5, 15, 30, 90min and 24h) in placebo (n=23) and cyclosporin-treated (n=24) patients with ST-segment elevation myocardial infarction (STEMI). *P<0.05 for each pair-wise analysis compared to the 24h time-point (Wilcoxon signed-rank test). Dunn’s multiple comparisons test between placebo and cyclosporin-treated patients for each time point are all non-significant.

The descriptive characteristics of the study population are reported in **Table 1**. IP-10 levels were determined during the acute phase of STEMI at admission before primary PCI-mediated reperfusion as well as at various sequential time points post-reperfusion (5, 15, 30, 90min and 24h). We observed that serum levels of IP-10 peaked at 15min after reperfusion, followed by an approximately 0.5-fold decrease at 90min post-reperfusion compared to admission levels (**Figure 2A**). IP-10 concentrations reached their lowest point after 24 hours (**Figure 2A**).

The primary objective of the “CAPRI” trial was to determine whether administration of cyclosporine before primary percutaneous coronary intervention (PPCI) reduces the amount of damage to the heart relative to treatment with placebo. We, therefore, assessed whether cyclosporin treatment affected IP-10 levels. We found that IP-10 kinetics were remarkably similar in the cyclosporin and placebo group (**Figure 2B**) and pair-wise analysis at each time point showed that cyclosporin did not affect IP-10 levels at any studied time point. Therefore, we used the whole study cohort for subsequent analyses, and we adjusted for randomization treatment where applicable.

### 3.2 Dynamics of T cell response in STEMI Patients

**Figure 3:**
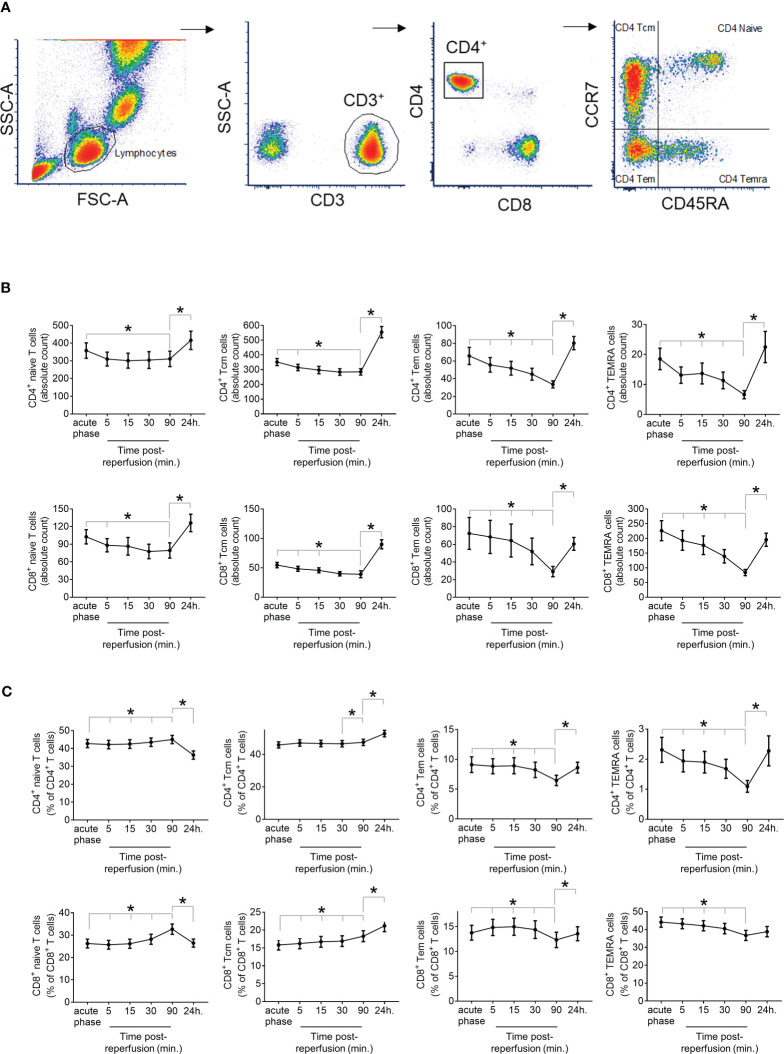
Dynamics of T cell response in STEMI Patients. **(A)** Immunophenotyping of the T-lymphocyte subtypes CD4^+^ or CD8^+^ naïve T cells, central memory T cells (T^cm^), effector memory T cells (T_EM_), or effector memory T cells re-expressing CD45RA (T_EMRA_) was based on the expression of CD45RA and CCR7 within CD4^+^ and CD8^+^ subsets of CD3^+^ peripheral blood mononuclear cells determined by 6-colour flow cytometry. **(B)** Absolute count presented at cells/µl of correctly peripheral blood and **(C)** Percentage of lymphocyte subset at admission (acute phase) and at sequential time-points post-reperfusion (5, 15, 30, 90min and 24h) in 47 STEMI patients. *P<0.05 for time-point comparison by Wilcoxon signed-rank test.

IP-10 is a major chemotactic factor for activated T-cells in inflamed tissues and is a homing chemokine for T cells that express CXCR3 (30). We first establish the kinetics of T cell subsets in fresh blood samples from STEMI patients, drawn at different time points. Using multicolour flow cytometry, we quantified the absolute count and the percentage of CD4^+^ and CD8^+^ subsets, including T_Naive_, T_CM_, T_EM_ and T_EMRA_ within the total CD4^+^ and CD8^+^ cells (**Figure 3A**). We observed a similar trend of the overall kinetics between CD4^+^ and CD8^+^ subsets. All cell subset counts were lower at 90 min compared with the admission levels, indicating they dropped during the first 90min post coronary revascularization (primary percutaneous coronary intervention). The 90 min drop observed in CCR7^-^ effector memory cells compared to admission was more drastic than in CCR7^+^ subsets (CD4^+^ T_EM_: 48.8%, CD4^+^ T_EMRA_: 64.3%, CD8^+^ T_EM_: 59.7%, and CD8^+^ T_EMRA_: 62.3% vs CD4^+^ T_Naive_: 13.1%, CD4^+^ T_CM_: 18.9%, CD8^+^ T_Naive_: 22.7%, and CD8^+^ T_CM_: 28.4%) (**Figure 3B**). Similarly, CCR7^-^ effector memory cells percentage of CD4^+^ or CD8^+^ T cells decreased at 90 min compared to admission, while the percentage of CCR7^+^ subsets increased slightly (**Figure 3C**). Furthermore, all counts of T cell subsets were all significantly higher at 24 hours compared to 90 min (**Figure 3B**).

### 3.3 IP-10 levels are associated with effector memory CD4^+^ T cell levels in blood

**Figure 4:**
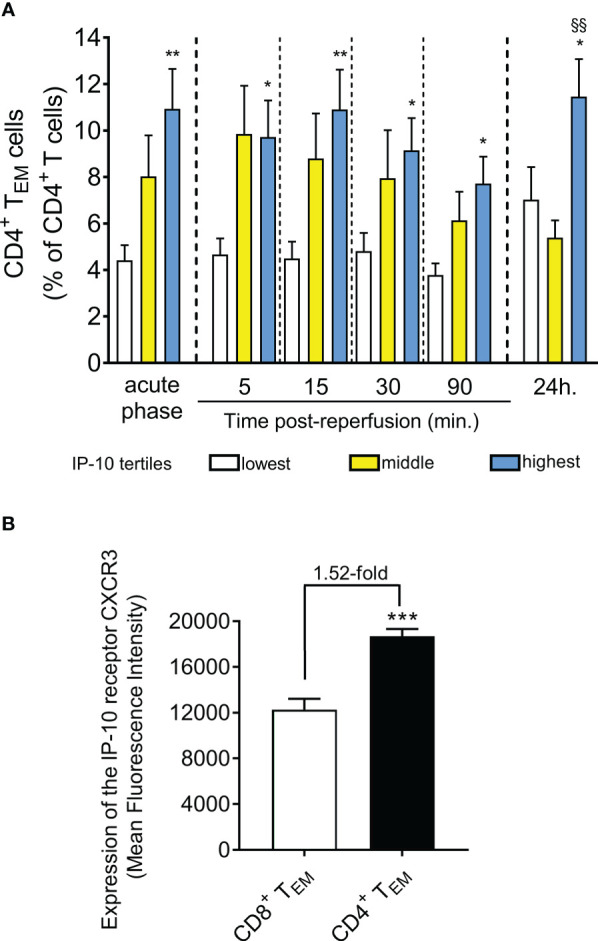
STEMI patients with higher IP-10 serum levels are presented with higher effector CD4^+^ T cell levels in blood. **(A)** Levels of CD4^+^ T_EM_ cells are shown based on IP-10 serum tertiles at admission (acute phase) and five time-points post-reperfusion. n=47, ^*^P<0.05 and ^**^P<0.01 compared to the lowest tertile, ^§§^P<0.01 compared to the middle tertile by Mann Whitney U test. **(B)** Expression of the IP-10 receptor CXCR3 was determined by flow-cytometry in CD4^+^ and CD8^+^ T_EM_ cells derived from 10 STEMI patients. ***P<0.001 by independent samples t-test.

In an attempt to provide a mechanistic explanation for the observed differences in the temporal T cell responses, we examined the potential differences in CD4^+^ or CD8^+^ T cell subtypes. Remarkably we observed that patients at the highest IP-10 tertile have a significant increase of CD4^+^ effector memory T cells (CD4^+^ T_EM_) within the CD4^+^ T cell compartment compared to the lowest tertile (**Figure 4A**). In contrast, there was no clear association between serum IP-10 levels and the percentage of CD8^+^ effector memory T cells (CD8+ T_EM_) within the CD8^+^ T cell compartment in blood. No association was observed between IP-10 serum levels and the other CD4^+^ or CD8^+^ T cell subtypes across all time points (data not shown).

To understand the positive association of IP-10 levels specifically with CD4**^+^** T_EM_, we quantified the levels of the IP-10 receptor CXCR3 on CD4^+^ and CD8^+^ T_EM_ cells. Interestingly, we found that IP-10 homing receptor CXCR3 expression was 1.52-fold higher on the surface of CD4^+^ T_EM_ cells compared to CD8^+^ T_EM_ cells (**Figure 4B**) suggesting that CD4**^+^** T_EM_ cells are more responsive to IP-10 changes in blood.

### 3.4 IP-10 levels predict recovery of left ventricular systolic function 12 weeks after STEMI

**Figure 5:**
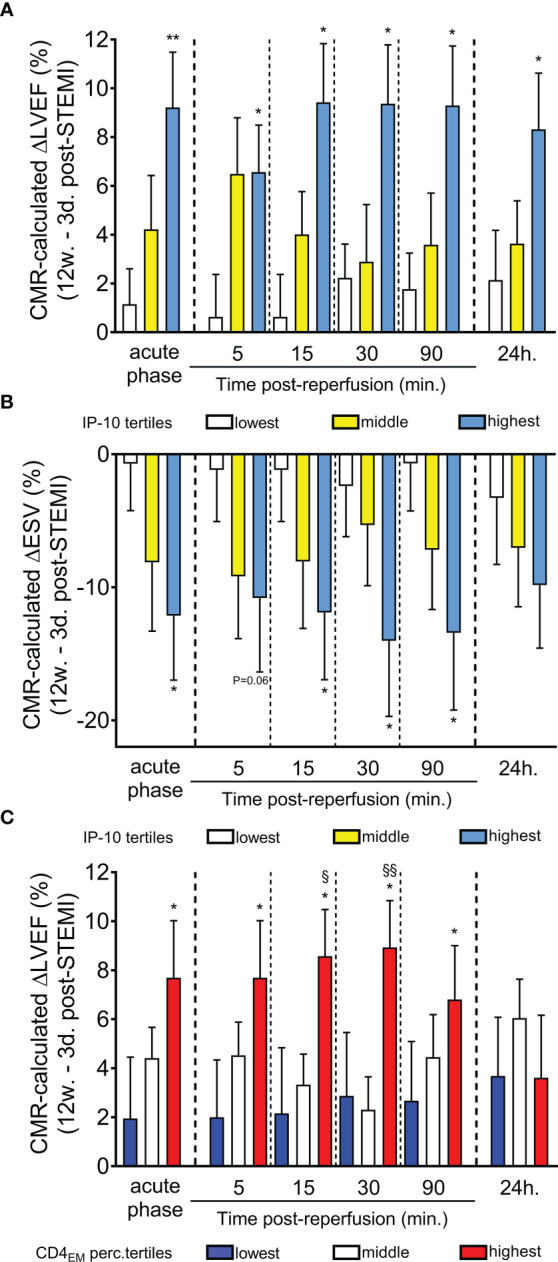
Predictive value of serum IP-10 levels and circulating CD4^+^ T_EM_ cells for recovery of left ventricular systolic function post-STEMI. **(A)** Cardiac magnetic resonance imaging-based ΔLVEF (12-week LVEF – 3-day LVEF) and **(B)** ΔESV (12-week ESV – 3-day ESV) is shown according to IP-10 serum tertiles at admission (acute phase) and at five time-points post primary percutaneous coronary intervention. ^*^P<0.05 and ^**^P<0.01 compared to lowest tertile by Mann Whitney U test. **(C)** CMR-based ΔLVEF (12-week LVEF – 3-day LVEF) is shown at six time-points based on CD4^+^ T_EM_ cell tertiles at admission. n=47, ^*^P<0.05 compared to the lowest tertile, ^§^P<0.05 and ^§§^P<0.01 compared to the middle tertile by Mann Whitney U test.

We then examined the relationship between IP-10 tertiles at admission and left ventricular systolic function improvement by calculating the change (Δ) in LV ejection fraction (LVEF), a predictor of heart failure (31–33), as determined by CMR imaging at 12 weeks minus the LVEF obtained 2-7 days after STEMI (ΔLVEF= LVEF at 12 weeks – LVEF at 2-7 days). Remarkably, high IP-10 levels on admission (acute phase) and within the first 24h after STEMI were associated with left ventricular function recovery 12 weeks after STEMI (**Figure 5A**). We also estimated the relationship between IP-10 tertiles at admission and end-systolic volume (ESV), another predictor of heart failure (34–36). We estimated the ESV improvement by calculating the change (Δ) in ESV as determined by CMR imaging at 12 weeks minus the ESV obtained 2-7 days after STEMI (ΔESV= ESV at 12 weeks – ESV at 2-7 days). High IP-10 levels on admission and within the first 24h after STEMI were associated with end-systolic volume decrease 12 weeks after STEMI, suggesting that the dynamic biphasic regulation of IP-10 in blood at the acute phase of myocardial infarction possibly confers a cardioprotective role (**Figure 5B**).

To examine the association of continuous IP-10 levels with myocardial function improvement (ΔLVEF), we used multivariable linear regression analysis adjusting for randomized intervention (cyclosporine vs. placebo), age, sex, body mass index, traditional cardiovascular risk factors including diabetes mellitus, arterial hypertension and hypercholesterolemia, as well as high-sensitivity cardiac troponin T (hs-cTnT) (**Table 3**). We found that IP-10 levels at admission were independently associated with higher ΔLVEF after adjustment of all these factors.

**Table 3:** Association of continuous IP-10 levels with ΔLVEF 12 weeks after STEMI in the Newcastle cohort by linear regression analysis.

Additional variables in the model	Coefficient (95% C.I.)* for IP-10	P-value for IP-10
Univariate	0.037 (0.008/0.067)	0.015
Age, sex	0.032 (0.002/0.063)	0.040
BMI, DM, hypertension, hyperlipidemia, eGFR	0.046 (0.012/0.079)	0.009
Admission hs-cTnT**	0.037 (0.006/0.067)	0.019
Age, sex, BMI, DM, hypertension, hyperlipidemia, eGFR	0.040 (0.005/0.075)	0.027
Age, sex, BMI, DM, hypertension, hyperlipidemia, eGFR, admission hs-cTnT**	0.041 (0.005/0.076)	0.028
Age, sex, BMI, DM, hypertension, hyperlipidemia, eGFR, admission hs-cTnT**, cyclosporin allocated intervention	0.040 (0.005/0.075)	0.028

* β-coefficients are derived from linear regression analysis with ΔLVEF as dependent variable, and IP-10 levels at the acute phase plus the variables shown in each row as independent variables. β-coefficients (95% C.I.) correspond to per one unit increase for continuous variables or versus the reference category for categorical variables.

** β-coefficients (95% C.I.) correspond to 100pg/ml increase in hs-cTnT.

Cyclosporin allocated intervention: Following angiography, participants were randomised in a 1:1 ratio to either cyclosporin or control (saline) using a blocked allocation (permuted random blocks of variable length) system. Randomisation included stratification by infarct location (anterior or nonanterior) and sex.

BMI, body mass index; DM, diabetes mellitus; eGFR, estimated glomerular filtration rate; hs-cTnT, high-sensitivity cardiac troponin T.

Importantly and in a similar manner to IP-10 tertiles, patients at the highest tertile of CD4^+^ T_EM_ cells on admission had a higher left ventricular systolic function recovery 12 weeks after STEMI (**Figure 5C**). No association between CD8^+^ T_EM_ cells and cardiac recovery was found.

### 3.5 IP-10 levels on admission and risk for major cardiovascular outcomes in patients after STEMI

**Figure 6:**
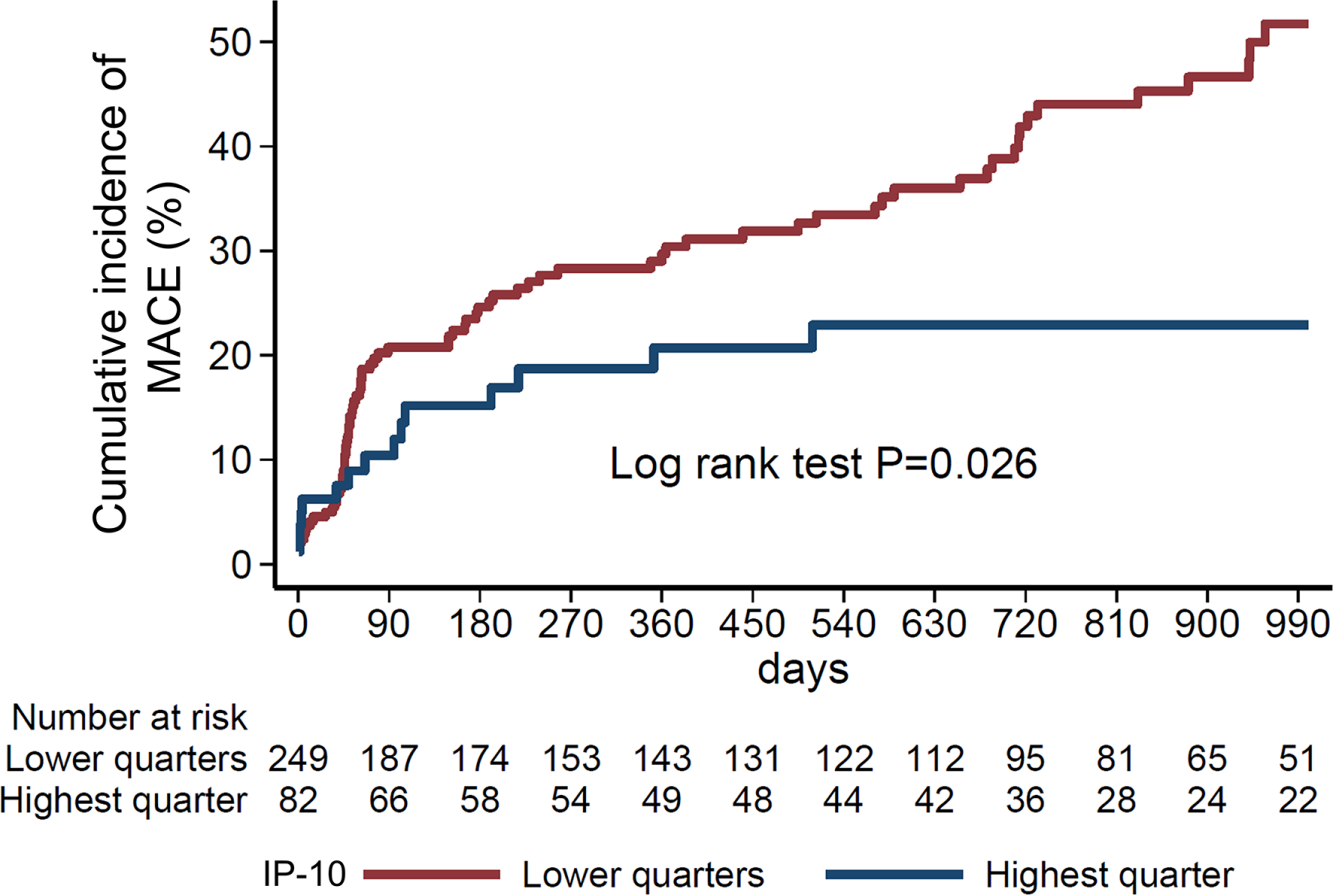
Nelson-Aalen curves of IP-10 quarters with the study endpoint. Cumulative incidence of the study endpoint (composite of all-cause death, myocardial infarction, stroke, resuscitation or revascularisation (percutaneous coronary angiography, coronary artery bypass graft) according to IP-10 quarters (highest vs. lower quarters) in 331 consecutive STEMI patients of the Heidelberg cohort. P=0.026 by Log-rank test.

We next evaluated the prognostic value of IP-10 for the prediction of major cardiovascular events (MACE) including death, new myocardial infarction, stroke, revascularisation (CABG or PCI) or resuscitation, in an independent cohort of 331 consecutive STEMI patients (**Figure 6**), as described in the methods (**Table 2**). Patients were followed over a median of 540 days for MACE, and the primary endpoint occurred in 105 (31.8%) patients. Patients at the highest IP-10 quarter at admission had reduced risk for the primary endpoint (HR=0.536 for highest vs lower quarters, 95% CI: [0.309-0.93], p=0.027 by Cox regression analysis, **Table 4**). After adjusting for age, sex, traditional cardiovascular risk factors (diabetes mellitus, arterial hypertension and hypercholesterolemia), eGFR, CRP and high-sensitivity troponin T levels at admission, we observed that higher IP-10 remained an independent predictor of lower incidence of MACE in STEMI patients (adjusted HR=0.420 for highest vs lower quarters, 95% CI: [0.218-0.808], p=0.009; **Table 4**).

**Table 4:** Association of continuous IP-10 levels with the study endpoint (MACE) in the Heidelberg cohort by Cox regression analysis.

Additional variables in the model	Hazard ratio (95% C.I.)*	P-value
Univariate	0.536 (0.309/0.930)	0.027
Age, sex	0.482 (0.276/0.842)	0.010
DM, hypertension, hyperlipidemia, eGFR	0.434 (0.229/0.834)	0.011
Admission hs-cTnT**	0.566 (0.325/0.985)	0.044
CRP**	0.505 (0.289/0.882)	0.016
Age, sex, DM, hypertension, hyperlipidemia, eGFR	0.416 (0.218/0.793)	0.008
Admission hs-cTnT**, CRP**	0.543 (0.311/0.950)	0.032
Age, sex, DM, hypertension, hyperlipidemia, eGFR, admission hs-cTnT**, CRP**	0.420 (0.218/0.808)	0.009

* Hazard ratios (HR) are derived from Cox regression analysis with the combined endpoint of all-cause death, resuscitation, myocardial infarction, stroke, CABG, PCI as dependent variable, and highest IP-10 quarter plus the variables shown in each row as independent variables. HR (95% C.I.) correspond to per one unit increase for continuous variables or versus the reference category for categorical variables.

** Hazard ratios (95% C.I.) correspond to 100pg/ml increase in hs-cTnT and 100mg/L in CRP.

DM, diabetes mellitus; eGFR, estimated glomerular filtration rate; hs-cTnT, high-sensitivity cardiac troponin T, CRP: C-reactive protein.

## 4 Discussion

**Figure 7:**
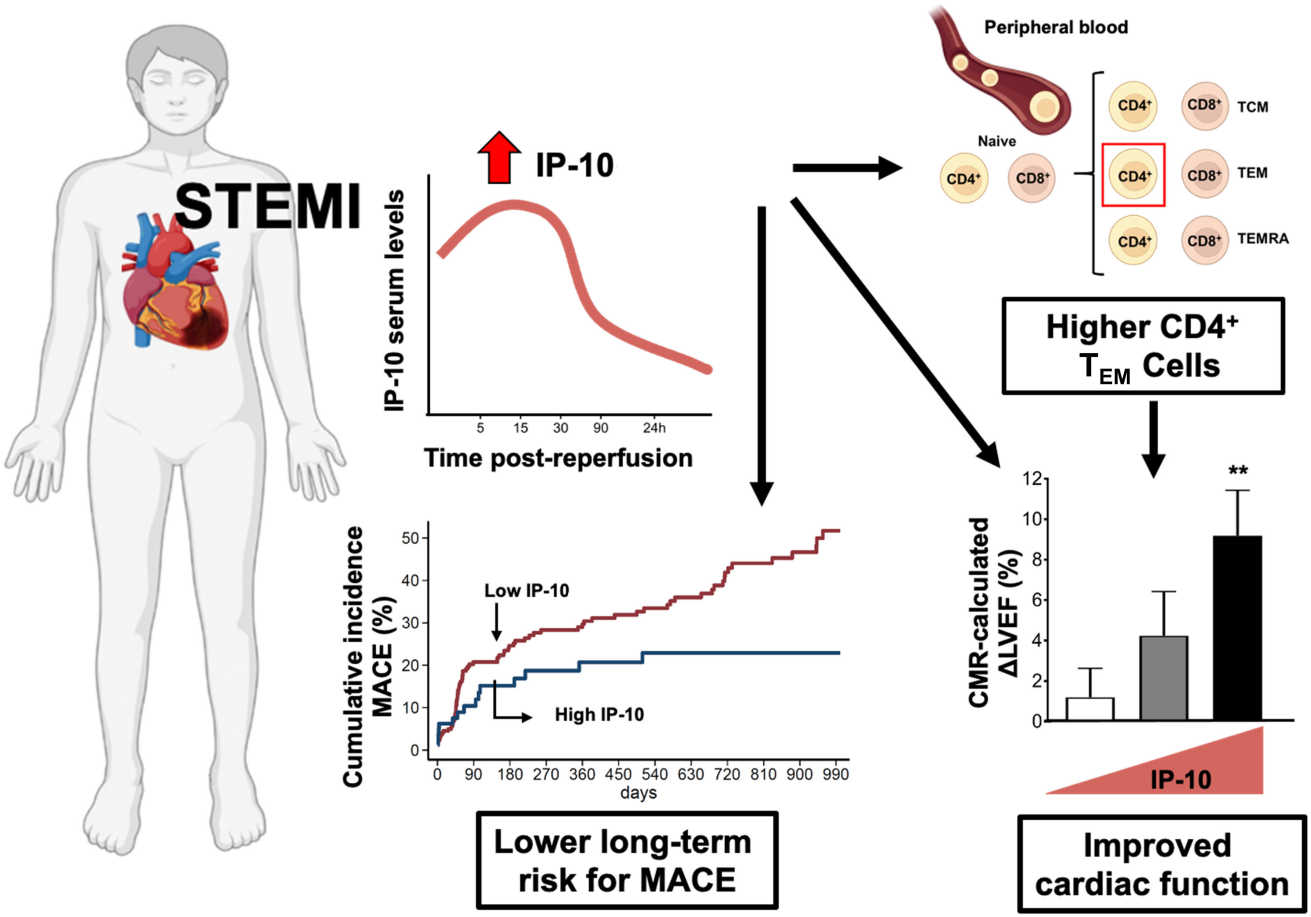
Summary figure of the potential role of IP-10 in STEMI patients. Effector T cell chemokine IP-10 levels predict recovery of left ventricular systolic function and cardiovascular outcomes after STEMI. IP-10 – CXCR3^+^ effector CD4^+^ T cells axis is critically involved in myocardial repair and overall disease prognosis after STEMI.

The major findings of the present study are: 1) increased serum IP-10 levels before and early after reperfusion are associated with improved cardiac systolic function in patients after STEMI; 2) increased serum IP-10 levels are associated with higher blood levels of CD4^+^ T_EM_ cells, but not other CD4^+^ or CD8^+^ cell subtypes; 3) higher CD4^+^ T_EM_ cells on admission are associated with LVEF increase 12 weeks after STEMI and 4) higher IP-10 serum levels on admission predict a lower long-term risk for MACE in patients with STEMI (**Figure 7**).

Chemokine release during acute myocardial infarction triggers the mobilisation of leukocytes, trafficking and domiciliation to ischemic tissues controlling wound healing processes, which critically determine cardiac repair post-myocardial infarction (37, 38). In the present study, we report a biphasic response of the effector T cell trafficking chemokine IP-10 to acute STEMI as follows: the serum IP-10 levels were high in the acute phase of STEMI (from patient admission to the hospital up to the first 15min post reperfusion of the occluded coronary artery), followed by a rapid profound reduction already by 90min post reperfusion. This is in accordance with previous preclinical animal data demonstrating that IP-10 peaks within 1–3 h of reperfusion and virtually disappears 24 h after reperfusion (19). This biphasic response may reflect the myocardial tissue ischemia level in STEMI. The initial high myocardial tissue ischemia caused by the infarcted coronary artery is followed by heart tissue reoxygenation after the reperfusion of the coronary circulation of the infarcted coronary artery following PPCI. This IP-10 early rise during acute ischemia indicates that hypoxia may trigger the increased serum levels of IP-10 chemokine. Indeed, the IP-10 gene promoter contains binding sites for both HIF1α and NF-κB, leading to increased IP-10 expression in cardiac microvascular endothelial cells upon hypoxia or ischemia (39).

IP-10 is an essential component of the adaptive immune response regulating the clearance of necrotic tissue in the heart. Absence of IP-10 in mice subjected to experimental myocardial infarction results in a deficient inflammation resolution and adverse cardiac remodelling (20). IP-10 knockout mice exhibit early dilation, followed by a rapid wall thinning during infarct maturation leading to systolic dysfunction and delayed scar contraction (20). In accordance with these preclinical data, we report that high levels of IP-10 during the acute phase of a myocardial infarction predict cardiac repair defined as the increase of LVEF and a decrease of ESV 12 weeks after STEMI, as quantified by cardiac MRI. Importantly, high IP-10 levels on admission predict a lower risk for MACE in an independent cohort of consecutive patients with STEMI. These findings support the notion that high IP-10 in the early acute phase of STEMI is integral for the reparative response in STEMI. Our study suggests that measurement of IP-10 serum levels may serve as a predictor of cardiac outcomes in STEMI patients.

IP-10 is a chemokine involved in the recruitment of activated T cells into sites of tissue inflammation (40). However, T cell temporal and spatial dynamics following myocardial ischemia and their contribution to inflammation resolution remain poorly understood. We have previously reported that T cells are specifically depleted from peripheral blood during myocardial reperfusion and lymphopenia is a critical determinant of survival in patients with STEMI (8, 22, 41). Here we conducted immunophenotyping of T cells at admission and within the first 24 hours post-reperfusion. Our results confirmed a rapid depletion of activated circulating CD4^+^ and CD8^+^ T cell subsets from admission to 90 minutes post-reperfusion in STEMI patients. This fall is probably followed by a rapid proliferative response in all T cell subsets after 24 hours, as suggested by previous studies in mice (9, 42). T effector memory cells are known for their migratory ability to inflamed tissue and the release of potent inflammatory mediators (43). In our study, these cells were drastically depleted from peripheral blood during the first 90 minutes of reperfusion, suggesting a potential transmigration of these cells to the infarcted heart during reperfusion. Our findings are in agreement with previous pre-clinical murine disease models showing that during ischemia/reperfusion, CD4^+^ and CD8^+^ T cells rapidly transmigrate to the infarcted areas of the heart (14, 42–46). CD4^+^ T cells become activated and are required for the clearance of necrotic tissue and subsequent remodeling of the myocardium through regulation of the local cytokine/chemokine environment (9, 10, 13, 47). On the other hand, CD8^+^ T cells may be largely detrimental to cardiac recovery post-MI by amplifying neutrophil and macrophage-mediated inflammation, thus resulting in increased left ventricle dilation and decreased cardiac function (13, 14). The increase in all T cell subsets after 24 hours in peripheral blood could be partly explained by the increased haematopoiesis observed after acute myocardial infarction and/or further mobilisation of lymphocytes from spleen, a secondary lymphoid organ acting as a T cell reservoir, lymph nodes or the bone marrow (48–52).

IP-10 homing receptor CXCR3 is the receptor with the highest expression in CD4^+^ and CD8^+^ T cells compared to other chemokine receptors (8). In our study, we report that CXCR3 expression is higher on CD4^+^ T_EM_ cells compared to CD8^+^ T_EM_ cells, suggesting that IP-10 promotes CXCR3^+^CD4^+^ T cell cardiotropism. Furthermore, the massive drop in IP-10 at 90 min is associated with falling CD4^+^ T_EM_ cells at 90min post reperfusion suggesting that the IP-10 response may reflect the levels of circulating CD4^+^ T_EM_ cells. Indeed, CXCR3^+^ T cells express higher levels of the integrin LFA-1, increasing their potential to adhere more efficiently to ICAM-1 and infiltrate the infarcted heart (53). Interestingly, IP-10 deficiency was associated with reduced recruitment of cell subpopulations that expressed CXCR3 (20). Although CXCR3 is highly expressed in CD8^+^ cells, it appears that the chemokine fractalkine and its receptor CX_3_CR1 are the driver of CD8^+^ T_EM_ cells dynamics in STEMI patients (8). Furthermore, the low expression of CXCR3 and CX_3_CR1 on naïve cells may explain the unique association in our study between IP-10 and CD4^+^ T_EM_ cells (8, 54). The data in our present study therefore highlights that several chemokine mechanisms are in play in the lymphocyte kinetics in ischemia-reperfusion.

Taken together, our data suggest that trafficking of CD4^+^ T_EM_ cells in response to acute myocardial ischemia may be orchestrated in an IP-10 – CXCR3 dependent manner controlling cardiac repair in AMI. This is in accordance with previous preclinical reports in animal models showing that CD4^+^ effector T cells are rapidly recruited into ischemic myocardium acquiring a cardioprotective phenotype (9, 10, 42–46). It is important to consider that the role of T cells in STEMI is likely time dependent. CD4^+^ T lymphocytes seem to exhibit biphasic kinetics post-myocardial infarction whereby CD4^+^ T cells facilitate wound healing in the acute phase of myocardial infarction but their further recruitment in the heart from the 10th day to 8 weeks after STEMI promotes left-ventricular dysfunction (55). IP-10 kinetics several weeks after STEMI and its contribution to this second wave, remains, however, to be determined. Given the observational, retrospective design of our study, our findings cannot prove causality. Nevertheless, our findings encourage further experimental research on the beneficial role of the increased blood levels of IP-10 in the acute phase of STEMI and the relative contribution of CD4^+^ T_EM_ cells in IP-10-related cardioprotective effects. There is therefore a need to further establish IP-10 as a prognostic marker in a larger STEMI cohort with heart failure as an end-point to relate individual IP-10 levels found in patients at admission to the risk of developing heart failure in the long-term.

In conclusion, the current study adds new translational evidence for the role of IP-10 in left ventricular function recovery and cardiovascular outcomes after STEMI. Understanding the differential effects of T cell subsets and their homing mechanisms to the ischemic heart is an essential first step for developing future targeted immunomodulatory therapies in patients with STEMI. Future studies are warranted to elucidate the underlying mechanisms and investigate the prognostic and therapeutic value of IP-10 modulation in patients with STEMI.

## Data availability statement

The raw data supporting the conclusions of this article will be made available by the authors, without undue reservation.

## Ethics statement

The studies involving human participants were reviewed and approved by NCT02390674. The patients/participants provided their written informed consent to participate in this study.

## Author contributions

Conception and design: IS, KSte. Analysis and interpretation of the data: KSo, ST-C, MM-H, NV, GG, EG, KB, AM, EG, IS, KSte. Drafting of the article: KSo, ST-C, IS, KSte. Critical revision of the article for important intellectual content: MM-H, NV, GG, MB, GR, EM, GR, AG, JH, HL, NF, KSta, JH, DD, EG. Provision of study materials or patients: MM-H, EG, IS. Statistical expertise: NV, GG, KSta. Obtaining of funding: KSo, ST-C, IS, KSte. Administrative, technical, or logistic support: MM-H, AT, MP-S, LS, KB. Collection and assembly of data: KSo, ST-C, MM-H, MB, MS, KB, AM. All authors contributed to the article and approved the submitted version.
